# Characterization of Duffy Binding Protein II-specific CD**4**^+^T cell responses in *Plasmodium vivax* patients

**DOI:** 10.1038/s41598-023-34903-4

**Published:** 2023-05-12

**Authors:** Pongsakorn Thawornpan, Chayapat Malee, Piyawan Kochayoo, Kittikorn Wangriatisak, Chaniya Leepiyasakulchai, Francis B. Ntumngia, Sai Lata De, John H. Adams, Patchanee Chootong

**Affiliations:** 1 Department of Clinical Microbiology and Applied Technology, Faculty of Medical Technology, Mahidol University, Bangkok, Thailand; 2 Center for Global Health and Infectious Diseases Research, College of Public Health, University of South Florida, Tampa, FL USA

**Keywords:** Infectious diseases, Cellular immunity

## Abstract

*Plasmodium vivax* Duffy Binding Protein region II (PvDBPII) is a leading vaccine candidate against blood-stage vivax malaria. Anti-PvDBPII antibodies potentially block parasite invasion by inhibition of erythrocyte binding. However, knowledge of PvDBPII-specific T cell responses is limited. Here, to assess the responses of PvDBPII-specific CD4^+^T cells in natural *P. vivax* infection, three cross-sectional studies were conducted in recovered subjects. In silico analysis was used for potential T cell epitope prediction and selection. PBMCs from *P. vivax* subjects were stimulated with selected peptides and examined for cytokine production by ELISPOT or intracellular cytokine staining. Six dominant T cell epitopes were identified. Peptide-driven T cell responses showed effector memory CD4^+^T cell phenotype, secreting both IFN-γ and TNF-α cytokines. Single amino acid substitutions in three T cell epitopes altered levels of IFN-γ memory T cell responses. Seropositivity of anti-PvDBPII antibodies were detected during acute malaria (62%) and persisted up to 12 months (11%) following *P. vivax* infection. Further correlation analysis showed four out of eighteen subjects had positive antibody and CD4^+^T cell responses to PvDBPII. Altogether, PvDBPII-specific CD4^+^T cells were developed in natural *P. vivax* infections. Data on their antigenicity could facilitate development of an efficacious vivax malaria vaccine.

## Introduction

*Plasmodium vivax* Duffy Binding Protein region II (PvDBPII), which binds to the Duffy antigen receptor for chemokines (DARC) on erythrocytes, is essential for parasite invasion. Given the ability to induce antibodies that block parasitic invasion into red blood cells, PvDBPII is considered as a promising blood-stage vaccine candidate. The major challenge of DBPII-based vaccine development is the highly polymorphic nature of this antigen. Following infection, early immune responses against DBPII are usually typified by strain-specific antibodies, and strain-transcending immunity can be elicited by repetitive exposure^1,2^. Development and maintenance of such inhibitory antibodies are observed in some individuals, suggesting that PvDBPII epitopes help elicit immunological memory^3,4^.

To develop vaccines targeted to conserved neutralizing DBPII epitopes, two approaches are proposed: (1) production of a single-allele antigen which is highly conserved across all DBPII variants; (2) production of a multi-allele antigen encompassing the major haplotypes found in malaria endemic areas. It was previously shown that both the DEKnull-based vaccine (PvDBPII without the dominant variant B-cell epitope) and the mixed-PvDBPII allele vaccine can confer neutralizing antibody responses ^5,6^. Strikingly, the mixed-allele approach helps generate inhibitory antibodies against a broader range of *dbpII* alleles ^5^. Further optimization will be required to enhance efficacy.

A better understanding of the generation and persistence of PvDBPII immunity is valuable for development of vaccines with long-term protection. Until now, the details of cellular immunity to PvDBPII are lacking. It is possible that T cell responses contribute to promotion of persistent inhibitory antibody responses to this antigen. Previous study in a highly endemic area of PNG demonstrated the generation of T cell immune responses to PvDBPII-derived peptide epitopes located in the binding motif of DARC ^6^. The production of PvDBPII-induced IFN-γ and IL-10 is greater in semi-immune adults compared to children < 5 years old, indicating that cellular immunity to PvDBPII may account for the development of acquired immunity^6^. However, there is a lack of studies describing which T epitopes are crucial for induction of protective immunity, and whether or not they trigger T cell responses that help B cell production of antibody. Furthermore, more investigation of cellular immunity against PvDBPII in endemic areas which vary in malaria transmission intensity could broaden knowledge regarding DBPII peptide-induced memory T cell responses, providing a stronger foundation for efficacious vaccine design.

In this study, we aimed to demonstrate which epitopes of PvDBPII induce development and persistence of cellular immunity in *P. vivax* infections. The potential DBPII-derived T cell epitopes were identified by in silico prediction of MHC class II-peptide binding and were synthesized for phenotypic and functional characterization of their ability to induce memory CD4^+^T cell responses. Our findings will hopefully guide further development of an efficacious PvDBPII vaccine, comprised of T cell epitopes, which are capable of stimulating memory CD4^+^T cell responses that can in turn facilitate durable protective immunity against vivax malaria.

## Methods

### Ethics statement

This study was approved by Mahidol University Central Institutional Review Board (MU-IRB 2012/079.2408 and MU-CIRB 2021/281.2505). Written informed consent was obtained from each participant before blood collection. All experiments involving human subject were conducted in accordance with relevant guidelines and regulations.

### Study design and cross-sectional surveys

**Figure 1:**
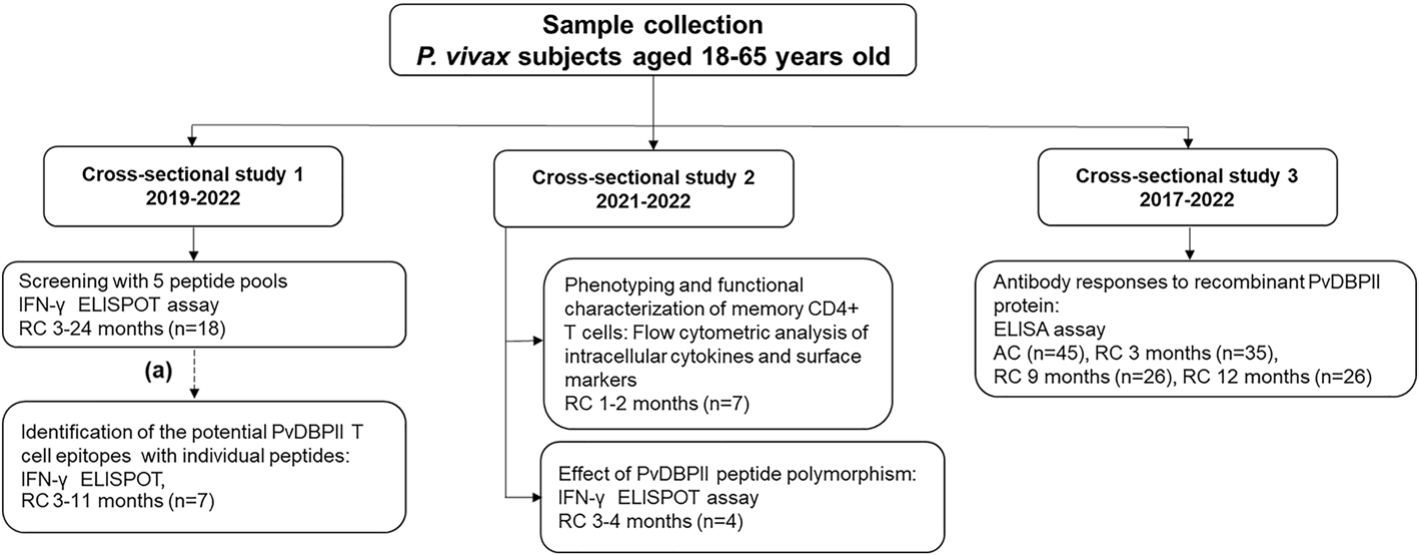
Study design. Schematic shows the work flow of three cross-sectional groups. Dashed line refers to samples which were taken for further experiments. (a) Subjects whose numbers of IFN-γ-secreting cells were higher than doubling of mean plus 2SD of spots in non-stimulated wells of *P. vivax* infected subjects were taken for further experiment as shown in arrow line. *AC* acute phase, *RC* recovery phase.

Heparinized blood samples were taken from subjects in malaria low-transmission areas in the southern part of Thailand (Chumphon and Ranong Provinces). A population-based open cohort study was carried out between 2017 and 2022, and included three cross-sectional surveys (Fig. 1). In cross-sectional study 1 (2019–2022), *P. vivax* subjects who had recovered from infection for 3–24 months (RC 3–24 m, n = 18) were enrolled for screening with PvDBPII peptide pools capable of stimulating memory T cell responses. To identify T cell epitope peptides, RC 3–11 m subjects (n = 7) who had a positive response (higher than cut-off value calculated from doubling of mean plus 2SD of spots in non-stimulated wells of *P. vivax* infected subjects) of IFN-γ-secreting cells in epitope screening experiment were selected. In cross-sectional study 2 (2021–2022), subjects recovered for 1–2 months (RC 1–2 m, n = 7) or for 3–4 months (RC 3–4 m, n = 4) were enrolled to analyze phenotypic and functional characteristics of PvDBPII-specific memory CD4^+^T cells, and to study the effect of polymorphism in PvDBPII sequence on IFN-γ response, respectively. In cross-sectional study 3 (2017–2022), to assess antibody response against PvDBPII, *P. vivax* subjects during acute malaria illness (n = 45) and after recovery from infection for 3 months (n = 35), 9 months (n = 26) or 12 months (n = 26) were enrolled. All recruited *P. vivax* subjects were infected for the first time without repeated malaria infection. Demographic information of recruited individuals is summarized in Supplementary Table S1 and S2.

Histories of prior malaria infections in individual subjects were obtained from the records of the malaria clinic at Vector Borne Disease Units 11.4.2 and 11.5.3. All *P. vivax*-infected subjects were examined microscopically by both thin and thick Giemsa-stained blood smears, and confirmed by nested PCR ^7^. Subjects were scheduled for blood sample collection every three months to assess possible sub-patent malaria by nested PCR. Staff conducted weekly house to house visits to estimate the incidence of clinical malaria over the study period. Blood samples from ten individuals who had no history of malaria infection were collected and used as healthy controls (HCs).

### In silico prediction of PvDBPII-derived T cell epitope peptides

The potential HLA class II binding peptides were predicted from the complete set of 15-mer peptides derived from PvDBPII-Sal I strain containing 330 amino acid residues (Supplementary Table S3**).** The length of synthesized peptides was set at 15 mers such that they stimulated naturally processed peptides by MHC class II molecules consisting of 9–14 residues ^8^. Three of the most prevalent MHC-II alleles in Thailand were used to predict the respective peptides, specifically HLA-DRB1*12:02 (15.32%), DQA1*01:01 (24.89%) and DQB1*05:02 (21.28%) ^8^. T-cell epitope prediction was performed by IEDB recommended version 2.2 (http://tools.iedb.org/mhcii/) ^9^. To identify peptides with highest binding to prevalent alleles of MHC class II, the predicted peptides were ranked on the basis of percentile rank scores; peptides with the lowest percentile scores were defined as the strongest MHC class II-binding peptides. From our analysis, 29 peptides with the lowest percentile ranks from prediction, and could be dissolved in DMSO and water, were selected for further immunological characterization.

### Synthesis of PvDBPII peptides

To identify principal T cell epitopes, a total of 29 DBPII-derived peptides were synthesized (each peptide was 15 mer in length). The 29 peptides were divided and mixed into five pools (pools A-E). Pools A-D contained six peptides each: Pool A (peptides 1, 2, 3, 4, 5 and10), Pool B (peptides 8, 12, 18, 20, 21 and 22), Pool C (peptides 7, 9, 14, 16, 17 and 19), Pool D (peptides 6, 11, 13, 15, 28 and 29). Pool E contained five peptides (peptides 23, 24, 25, 26 and 27)**.** Peptide 29 was reported to stimulate strong INF-γ responses in a previous study^6^. All peptides were custom-synthesized by GenScript. Peptide synthesis was done at 4 mg scale with an estimated purity of > 80.0% by the supplier (Supplementary Table S4).

In addition, to study the effect of PvDBPII polymorphism on IFN-γ responses, a set of peptides corresponding to the PvDBPII variants found to be widespread in malaria endemic areas of Thailand and other endemic areas, including Myanmar and Papua New Guinea)^1^ were synthesized. From six potential T cell epitope peptides, there were three (peptides 5, 13 and 18) that contained polymorphic sites of PvDBPII. Accordingly, peptides with polymorphic residues at R308S of peptide 5, L333F of peptide 13 and W437R of peptide 18 were synthesized.

### IFN-γ ELISPOT assay

Cryopreserved peripheral blood mononuclear cells (PBMCs) collected from *P. vivax* subjects were thawed and assessed if the viability was more than 95%. PBMCs at density of 1 × 10^6^ cells per well were stimulated at 37 °C for 36 h with five peptide pools (Pools A–E) or individual peptides (Supplementary Table S4), and 0.5 µg/ml of CD28/CD49d (BD Biosciences, USA) in 200 µl of R10 medium containing 10% fetal bovine serum (F7524, Sigma-Aldrich) in RPMI 1640 media and 1%penicillin/streptomycin. The concentration of each peptide in pool and individual platform was kept at 20 µg/ml. Multiscreen plates (Merck Millipore, Ireland) were coated with 15 µg/ml anti-human IFN-γ monoclonal antibody (1-D1K; Mabtech, Sweden). IFN-γ production was detected by 1 µg/ml of biotinylated anti-human IFN-γ monoclonal antibody (7-B6-1; Mabtech), 1:1000 streptavidin-ALP (Mabtech) and BCIP/NBT substrate (Mabtech). Positive controls were prepared by stimulation of PBMCs (2 × 10^5^ cells/well) with phytohaemagglutinin (PHA) at 2% v/v, while negative controls were prepared by PBMCs (1 × 10^6^ cells/well) without stimulation. PBMCs from ten healthy donors were used to determine baseline responses of stimulation and non-stimulation conditions. Positive IFN-γ responses to peptide pools were defined as responses that were higher than cut-off value calculated from doubling of mean plus 2SD of spots in non-stimulated wells of *P. vivax* infected subjects.

### Flow cytometric analysis of surface markers and intracellular cytokines.

To phenotype PvDBPII-induced CD4^+^ memory T cells, PBMCs (1 × 10^6^ cells/well) were stimulated for 18 h with a mixture of six potential epitope peptides; the concentration of each were prepared at 20 µg/mL in 200 µl of R10 medium. Cells were cultured at 37 °C, 5% CO_2_ for 6 h with 10 µg/mL Brefeldin A (Sigma). Following incubation, PBMCs were stained with Zombie green viability dye (Biolegend, USA). Antibodies specific to surface markers (CD3, CD4, CD45RA and CCR7) and intracellular cytokines (IFN-γ and TNF-α) were stained prior to flow cytometric analysis. Positive controls were prepared by stimulation of PBMCs (5 × 10^5^ cells/well) with 50 ng/ml of PMA (Sigma, USA) and 1 µg/ml ionomycin (Sigma) for 6 h, while negative controls were considered of PBMCs without stimulation. Details of antibody dilutions used in flow cytometry in this study are listed in Supplementary Table S5.

### IgG ELISA

Levels of anti-PvDBPII antibodies were determined by ELISA as previously reported^10^. Briefly, recombinant PvDBPII protein (rPvDBPII) at 2 µg/mL was coated on 96-well plates. Plasma (1:200 dilution) was added to wells followed by HRP-conjugated goat anti-human IgG (KPL, USA). Signal was developed with TMB substrate and read at 450 nm. Seropositive plasma from *P. vivax*-infected patients (n = 3), as characterized in a previous study ^10^, were used as positive controls; plasma from HCs (n = 23) were used as negative controls. Cut-off value was calculated from mean optical density (OD) values of these HCs plus 2SD. The antibody response was expressed as the reactivity index (RI) calculated by dividing mean OD value of each patient divided to cut-off value. Seropositivity is considered when RI value is greater than 1.0.

### HLA class II genotyping

To perform HLA genotyping, EDTA blood samples (3 ml) were collected from eight *P. vivax* subjects enrolled in cross-sectional study 1 and 2. Blood samples were submitted to perform high-resolution HLA class II genotyping at the Reference Laboratory Excellence Centre, National Blood Centre, Thai Red Cross Society (Bangkok, Thailand). Sequencing was performed using an Illumina sequencing platform with searching against IPD-IMGT/HLA Database version 3.48.0.0.

### Statistical analysis

Statistical analysis was performed using GraphPad Prism version 8.4.3, GraphPad Software, USA, https://www.graphpad.com. Statistical testing of peptide pool screening results was performed using Krustal-Wallis and Dunn’s multiple comparison tests. Statistical testing of the experiments of amino acid polymorphism effects was performed with a paired *t*-test. Statistical significance was considered as *p* < 0.05.

## Results

### In silico analysis of PvDBPII-derived T cell epitopes

**Figure 2:**
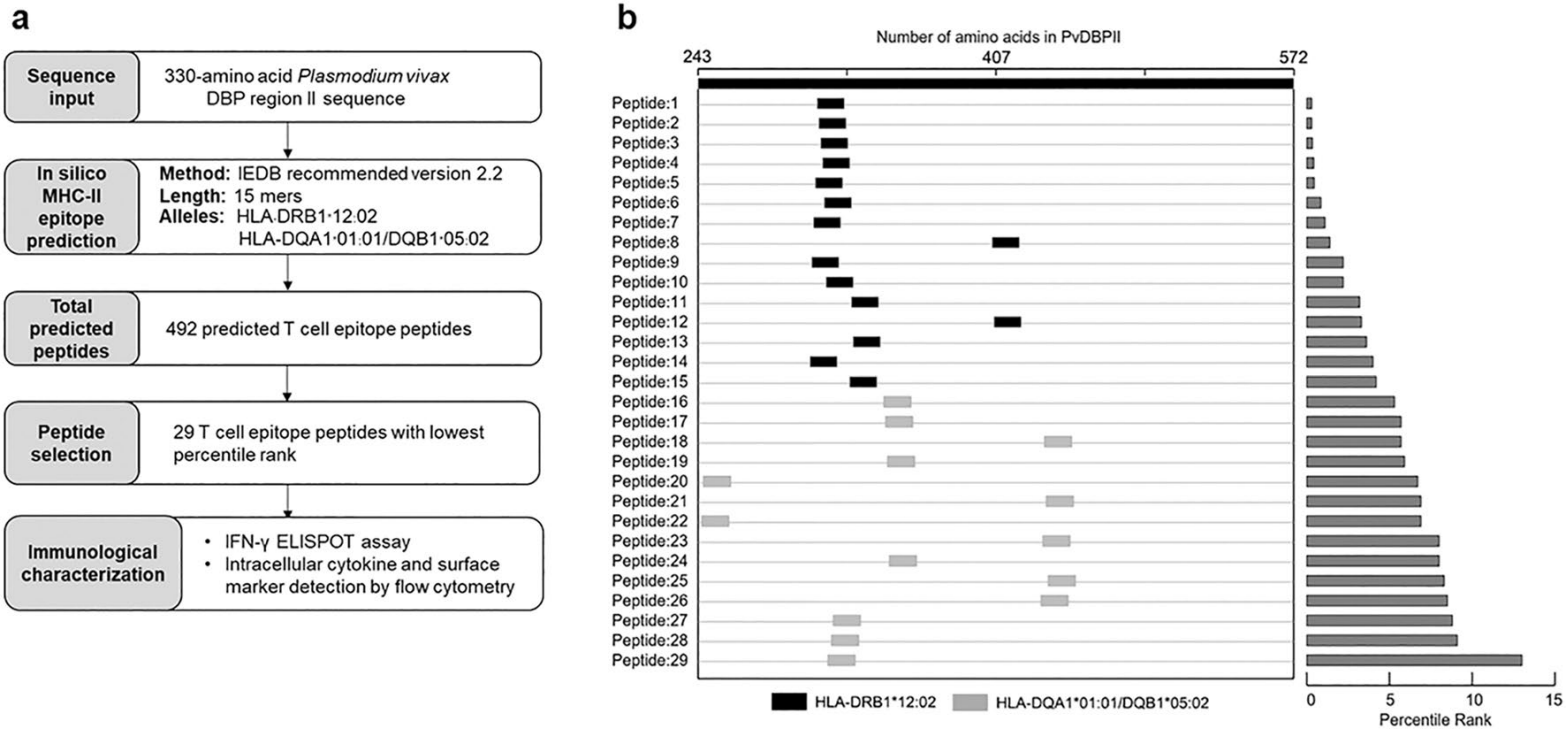
In silico prediction of DBPII T cell epitopes by the Immune Epitope Database (IEDB). (**a**) Prediction workflow and selection for peptides used for characterization of immune responses by immunological assays. (**b**) Relative location of twenty-nine 15-mer T cell epitopes with lowest percentile rank across the 330-amino acid sequence of PvDBPII (reference strain Sal I) from residue 243 to 572.

To perform in silico MHC class II-binding prediction, the 330-amino acid sequence of PvDBPII from reference strain Sal I was used as an input, and analyzed using the NetMHCIIPan algorithm embedded in The Immune Epitope Database (IEDB) (Fig. 2a). The prediction was done in the context of the two most common HLA class II alleles in the Thai population (HLA-DRB1*12:02 and HLA-DQA1*01:01/DQB1*05:02^7^. This in silico prediction gave a total of 492 putative PvDBPII-derived CD4^+^T cell epitopes, each consisting of 15 amino acids (Supplementary Table S6). From these predicted epitopes, 29 peptides with low percentile rank, corresponding to putative strong MHC class II-binding peptides, were shortlisted for peptide synthesis and further immunological characterization. The relative locations of these shortlisted CD4^+^T cell epitopes were mapped onto the sequence of PvDBPII template (Fig. 2b).

### Screening of PvDBPII peptide pools recognized by IFN-γ^+^ memory T cells

**Figure 3:**
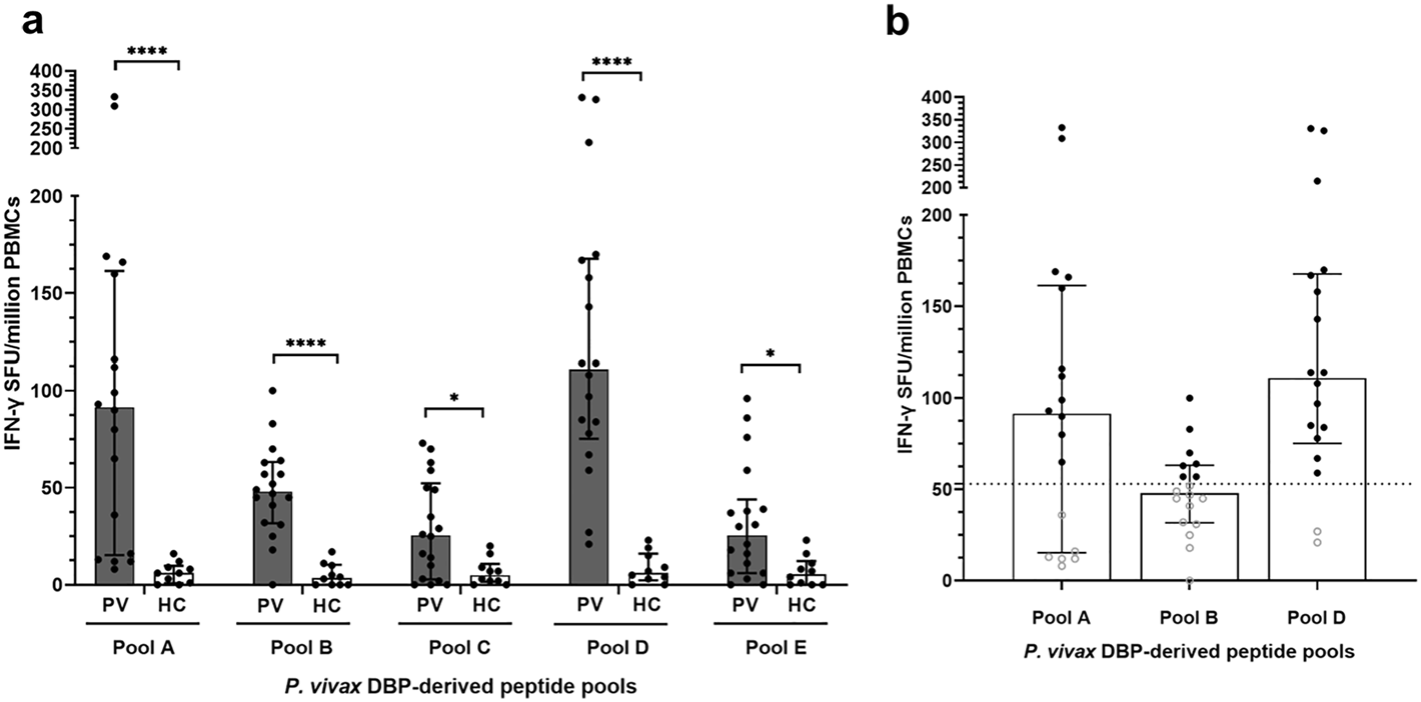
Number of IFN-γ producing cells after stimulation with five different *P. vivax* DBP-derived peptide pools in cross sectional study 1. (**a**) Net number of IFN-γ spot forming units (SFUs) per million PBMCs of recovered *P. vivax* subjects (PV, n = 18) after stimulation for 36 h with one of five distinct peptide pools compared to malaria naïve healthy controls (HC; n = 10). Net SFUs were calculated from a total number of spots in stimulated well minus background spots in non-stimulated well. Statistical testing used Krustal-Wallis test and Dunn’s multiple comparison test; *p < 0.05 and ***p < 0.0001. **(b)** Number of IFN-γ spot forming units (SFUs) per million PBMCs of recovered *P. vivax* subjects (PV, n = 18) after stimulation with pool A, pool B and pool D, which displayed significant difference in IFN-γ compared to malaria naïve subjects. Black dot represents positive response, while white spot represents negative response. Dashed line represents cut-off value (46 spots), calculated from doubling of mean plus 2SD of spots in non-stimulated wells of *P. vivax* infected subjects. Each bar represents median and error bar represents interquartile range (IQR). The experiments were performed in triplicate. Positive controls were prepared by stimulation of PBMCs (2 × 10^5^ cells/well) with phytohaemagglutinin (PHA, 2% v/v), while negative controls were prepared by PBMCs (1 × 10^6^ cells/well) without stimulation.

IFN-γ-producing cells stimulated by PvDBPII epitopes were detected after stimulation of PBMCs from *P. vivax* infected subjects (PVs) and malaria naïve healthy controls (HCs) with PvDBPII peptides (Supplementary Table S3). A total of 29 in silico-predicted PvDBPII-derived peptides (15-mers) were synthesized and grouped into five pools (Pools A–E). Compared to HCs, all *P. vivax* subjects showed significant responses of IFN-γ producing cells to all peptide pools: Pool A (median 91.5 [IQR 21–149]) vs. median 6 [IQR 1.5–8.75]), Pool B (median 48 [IQR 34.25–61.5) vs. median 3.5 [IQR 0–8.75]), Pool C (median 25.5 [IQR 4.75–49.75) vs. median 5 [IQR 2–8.5]), Pool D (median 111 [IQR 79.5–164.75 vs. median 6 [IQR 3.25–13.5]), Pool E (median 25.5 [IQR 7.25–38.75) vs. median 5.5 [IQR 0.25–10.25]). Representative INF-γ ELISPOT results were shown in Supplementary Fig. S1. Among these pools, there were three pools (pool A, pool B and pool D) that showed highly significant difference compared to HCs (Fig. 3a). Furthermore, pool A, pool B and pool D had median spots higher than spot cut-off value (46 spots) with seropositivity percentage of 66.7%, 55.6% and 88.9%, respectively (Fig. 3b). Thus, peptide Pools A, B and D were selected for immunological characterization in further experiments.

### Identifying T cell epitopes that induced IFN-γ memory T cell responses

**Figure 4:**
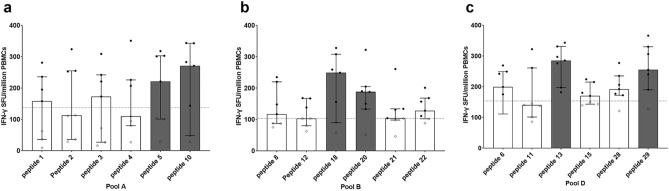
Number of IFN-γ producing cells after stimulation with individual peptides from each pool in cross sectional study 1. (**a–c**) Number of IFN-γ spot forming units (SFUs) per million PBMCs after stimulation with individual peptides from Pool A, Pool B and Pool D, respectively. PBMCs were taken from *P. vivax* subjects who had recovered for 3–11 months (n = 7) showing positive responses in peptide pool screening of PBMCs and were stimulated with individual peptides for 36 h. Gray bars represent individual peptides that induced the two highest medians of IFN-γ SFUs in each peptide pool. Dashed line represents cut-off values (137 spots for Pool A, 103 spots for Pool B and 153 spots for Pool D), calculated by doubling of mean plus 2SD of spots in non-stimulated wells of *P. vivax* infected subjects. Black dot represents positive response, while white spot represents negative response. Positive controls were prepared by stimulation of PBMCs (2 × 10^5^ cells/well) with phytohaemagglutinin (PHA, 2% v/v), while negative controls were prepared by PBMCs (1 × 10^6^ cells/well) without stimulation. The experiments were performed in triplicate.

To identify potential T cell epitopes in PvDBPII peptide pools, IFN-γ-secreting cells were detected using PBMCs from seven *P. vivax* subjects collected 3–11 months post-infection with or without stimulation by individual peptides contained in peptide Pools A, B and D. Having median spot numbers higher than doubling of mean plus 2SD of spots in non-stimulation condition (137 spots for Pool A, 103 for pool B and 153 for pool D), some peptides could remarkably stimulate higher number of IFN-γ-secreting cells: peptide 5 (RDITFRKLYLKRKLI) with median 221 (IQR 102.5–302) and peptide 10 (KLYLKRKLIYDAAVE) with median 271 (IQR 96–313) in Pool A, peptide 18 (YRWIREWGRDYVSEL) with median 249 (IQR 123–283.5) and peptide 20 (SAIINHAFLQNTVMK) with median 188 (IQR 141.5–197.5) in Pool B, as well as peptide 13 (LYLKRKLIYDAAVEG) with median 285 (IQR 236.5–314) and peptide 29 (GDLLLKLNNYRYNKD) with median 255 (IQR 215–318) in Pool D (Fig. 4a–c). Thus, these six peptides were identified as potential T cell epitopes driving memory T cell responses (Table 1).

**Table 1:** List of six potential PvDBPII-derived epitope peptides (15 mers) identified in this study.

Peptide no.	Peptide sequences	Residues
Peptide 5	RDITFRKLYLKRKLI	308–322
Peptide10	KLYLKRKLIYDAAVE	314–328
Peptide 13	GDLLLKLNNYRYNKD	329–343
Peptide 18	YRWIREWGRDYVSEL	435–449
Peptide 20	SAIINHAFLQNTVMK	246–260
Peptide 29	LYLKRKLIYDAAVEG	315–329

Next, we analyzed HLA allele-associated T cell responses, peptides in Pool D which showed high IFN-γ-producing cell response were further analyzed in five RC subjects (Supplementary Table S7).The HLA-DQA (A1*01:01/A1*03:03, A1*01:01/A1*01:05, A1*01:03/A1*03:02, DQA1*01:03/A1*06:01 and A1*01:04/A1*02:01), HLA-DQB (B1*04:01/B1*05:01, B1*05:01/B1*05:01, B1*03:03:02/B1*05:03:01, B1*03:01:01/B1*06:01:01 and B1*02:02/B1*05:03) and HLA-DRB (B1*04:05**/**B1:15:02, B1*01:01/B1*10:01, B1*04:05/B1*09:01, B1*08:03/B1*12:02, B1*07:01/B1*14:04) alleles in RC01-RC05 were allotypes that showed significantly increased IFN-γ responses to both peptides 13 and 29. The responses of IFN-γ producing cells to these two peptides suggested promiscuity of the HLA binding.

### Effector memory IFN-γ^+^CD4^+^T cells were activated by T cell epitopes

**Figure 5:**
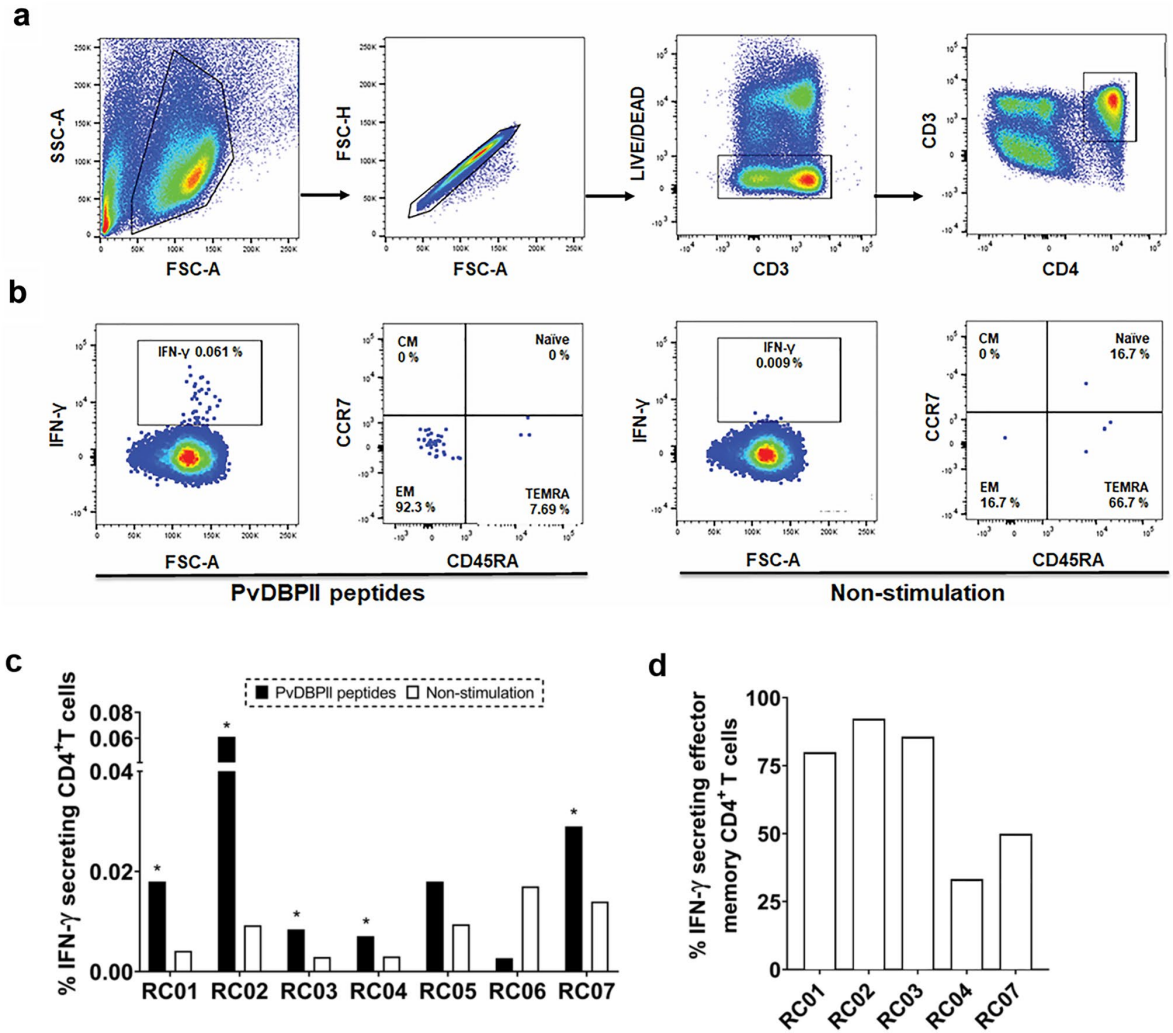
Frequency of IFN-γ-secreting cells after stimulation with 6 potential epitope peptides in cross-sectional study 2. (**a**) Gating strategy used to select live CD3^+^ CD4^+^ T cells. (**b**) A representative flow cytometric dot plot displaying IFN-γ-secreting CD4^+^T cells and corresponding memory T cell subsets without or with PvDBPII epitope peptide stimulation for 18 h using PBMCs (1 × 10^6^ cells/well) from *P. vivax* subjects. (**c**) Percentage of IFN-γ-secreting CD4^+^T cells after stimulation of PBMCs from *P. vivax*-infected subjects (RC01–RC07) recovered for 1–2 months with 6 potential epitope peptides. Positive responses were defined as percentage of IFN-γ-secreting CD4^+^T cells under peptide stimulation being at least twofold higher than that of non-stimulation condition. Asterisk (*) indicates positive response. (**d**) Percentage of IFN-γ-secreting effector memory CD4^+^T cells in *P. vivax*-infected subjects who displayed positive responses (RC01, RC02, RC03, RC04 and RC07). Positive controls were prepared by stimulation of PBMCs (5 × 10^5^ cells/well) with PMA (50 ng/ml) and ionomycin (1 µg/ml), while negative controls were prepared by PBMCs (5 × 10^5^ cells/well) without stimulation. *CM* central memory T cells, *EM* effector T cells, *TEMRA* effector memory T cells re-expressing CD45RA.

PvDBPII-specific T cells identified by IFN-γ production were characterized with established phenotypic markers of differentiation including lymph node homing receptor CCR7 and CD45RA expression (Fig. 5a, b), as previously described ^11^. Here, the flow cytometry plot clearly revealed that PvDBPII T cell peptides induced a higher number of IFN-γ secreting cells (0.061%) compared to the non-stimulated condition (0.009%) (Fig. 5b). Among seven studied subjects (RC01-07), levels of IFN-γ CD4^+^T cell responses were observed to be within the range of 0.003–0.061% (Fig. 5c). Based on positivity criteria (spots in stimulated well were two-fold higher than non-stimulated well), there were five subjects whose IFN-γ CD4^+^T cell responses were positive. The major T cell subset responsible for IFN-γ secretion consisted of effector memory T cells (CD3^+^ CD4^+^ CCR7^-^ and CD45RA^-^) which made up 33.3–92.3% (RC01 83.3%, RC02 97.4%, RC03 80.0%, RC04 33.3% and RC07 66.7%) of IFN-γ secreting CD4^+^T cell population after peptide stimulation (Fig. 5d).

### Effector memory CD4^+^T cells secreted IFN-γ and TNF-α in response to T cell epitopes

**Figure 6:**
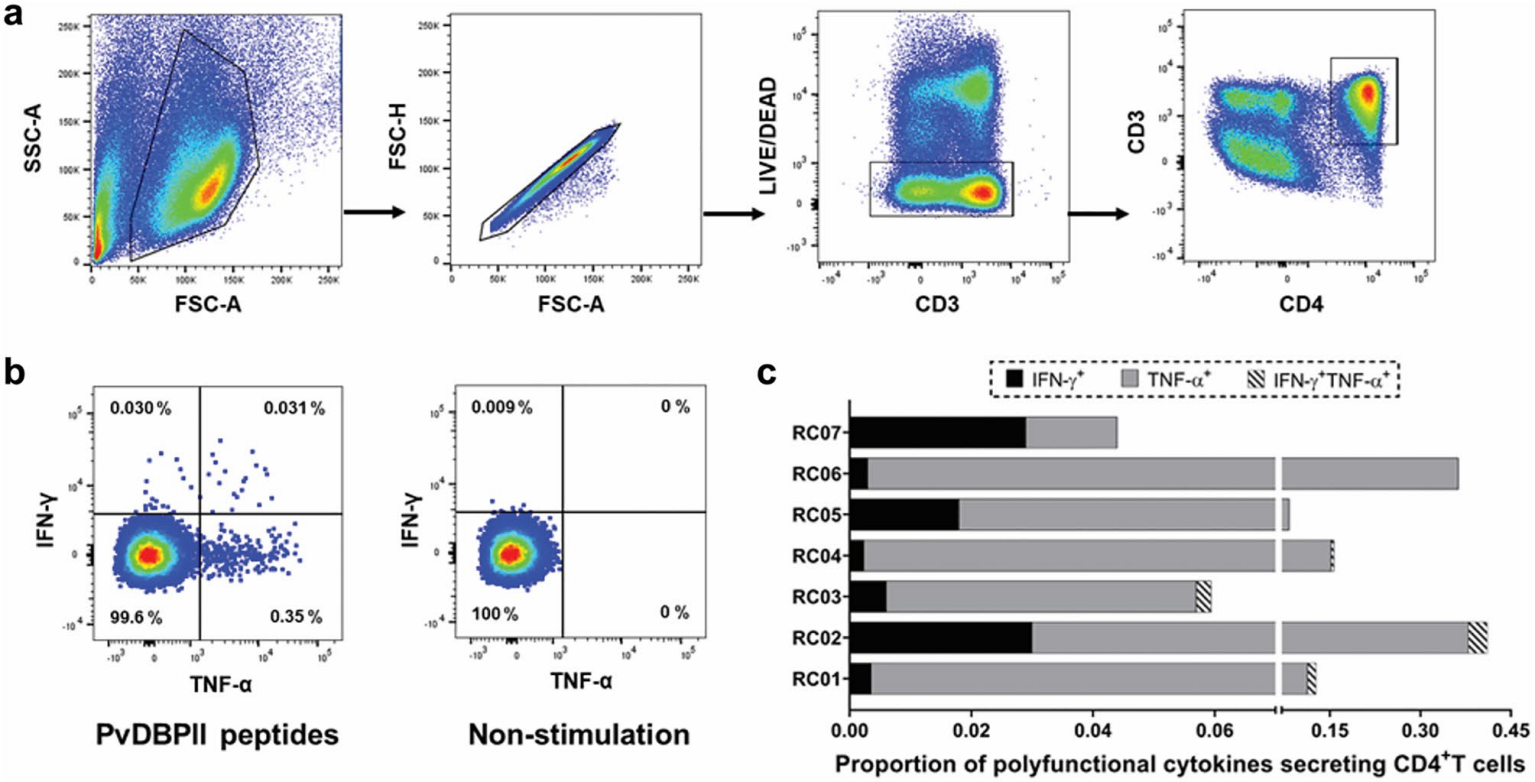
Polyfunctionality of memory T cells after stimulation with 6 potential epitope peptides in cross-sectional study 2. (**a**) Gating strategy used to select live CD3^+^CD4^+^T cells. (**b**) A representative flow cytometric dot plot displaying IFN-γ-, TNF-α secreting cells in conditions without or with six potential PvDBPII epitope stimulation. (**c**) Proportion of IFN-γ, TNF-α secreting CD4^+^T cells after stimulation of PBMCs from 1–2 months of recovery *P. vivax* subjects (n = 7; RC01–07) by 6 PvDBPII epitope peptides. PBMCs were stimulated with PvDBPII epitope peptides for 18 h.

To determine the potential polyfunctionality of cellular responses, T cells were evaluated for the secretion of IFN-γ and TNF-α cytokines in response to stimulation with six PvDBPII-derived T cell epitope peptides. Initially, PBMCs of *P. vivax* subjects recovered for 1–2 months (RC 1-2 m, n = 7) were stained and gated to select a population of live CD3^+^ CD4^+^T cells (Fig. 6a). After intracellular IFN-γ and TNF-α cytokines were stained, it was found that 57% (4/7) of studied subjects (RC01-RC04) harbored polyfunctional CD4^+^T cells that expressed IFN-γ and TNF-α, and whose frequency had a range of 0.002–0.031% (Fig. 6b, c). Of seven individuals, CD4^+^T cells that secreted only TNF-α^+^ varied from 0.015 to 0.350% while ones that secreted only IFN-γ^+^ varied from 0.002 to 0.030% (Fig. 6c). These data suggested the generation of a polyfunctional responses by memory CD4^+^T cells specific to PvDBPII peptides following *P. vivax* infection.

### PvDBPII polymorphisms affected IFN-γ^+^ memory T cell responses

**Figure 7:**
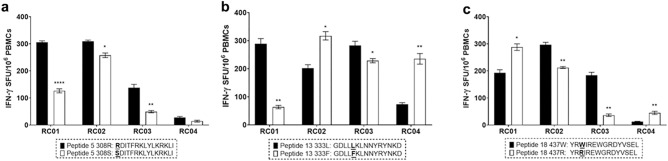
Effect of amino acid polymorphism of PvDBPII-derived peptides on interferon-γ response in cross-sectional study 2. Alteration of IFN-γ response in response to representative PvDBPII polymorphism was determined by a set of synthesized peptides (peptide 5 with 308R and 308S, peptide 13 with 333L and 333F, as well as peptide 18 with 437W and 437R). These peptides were designed by substitution of single amino acids corresponding to polymorphic sites of PvDBPII haplotypes identified in malaria endemic area in Thailand and other areas including Myanmar and Papua New Guinea. (**a–c**) Changes in the number of IFN-γ- spot forming units (SFUs) per million PBMCs after stimulation of PBMCs from four *P. vivax* recovered subjects (RC01–04) at 3–4 months post-infection with each polymorphic peptide for 36 h. Horizontal line represents mean; error bar represents standard deviation (SD). The experiment was performed in triplicate. Statistical testing was performed by paired *t*-test; *p < 0.05, **p < 0.005, ****p < 0.0001.

To examine whether PvDBPII polymorphisms affect the cellular responses induced by dominant T cell epitopes, we performed ex vivo stimulation of PBMCs from subjects recovered from *P. vivax* infection (RC01–04) at 3–4 months post-infection and compared the effect of amino acid substitution (Fig. 7). Three subjects displayed significant decrease (1.2–2.8-fold) in IFN-γ-secreting cell responses to peptide 5 with 308S variant (number of IFN-γ spots; RC01 = 126, RC02 = 258, RC03 = 49) compared to peptide 5 with 308R site (RC01 = 306, RC02 = 309, RC03 = 138) (Fig. 7a). For the 333F variant of peptide 13, lower frequencies of peptide-induced IFN-γ-secreting cells were detected in subjects RC01 (64 spots) and RC03 (229 spots), whereas subjects RC02 (317 spots) and RC04 (246 spots) responded significantly to this variant (Fig. 7b). Analysis of T cell responses to peptide 18 with 437R variant showed reduction of IFN-γ-secreting cell responses in subjects RC02 (212 spots) and RC03 (36 spots). However, subjects RC01 and RC04 generated strong IFN-γ producing T cell responses to 437R variant (RC01 = 288 spots, RC04 = 45 spots) (Fig. 7c). Of four subjects who displayed the increased IFN-γ responses to mutant peptides, HLA class II genotype was determined in three (Supplementary Table S8). The results showed that various alleles of HLA-DQA1, -DQB1 and -DRB1 were present in the three.

### Antibody responses against recombinant PvDBPII protein

**Figure 8:**
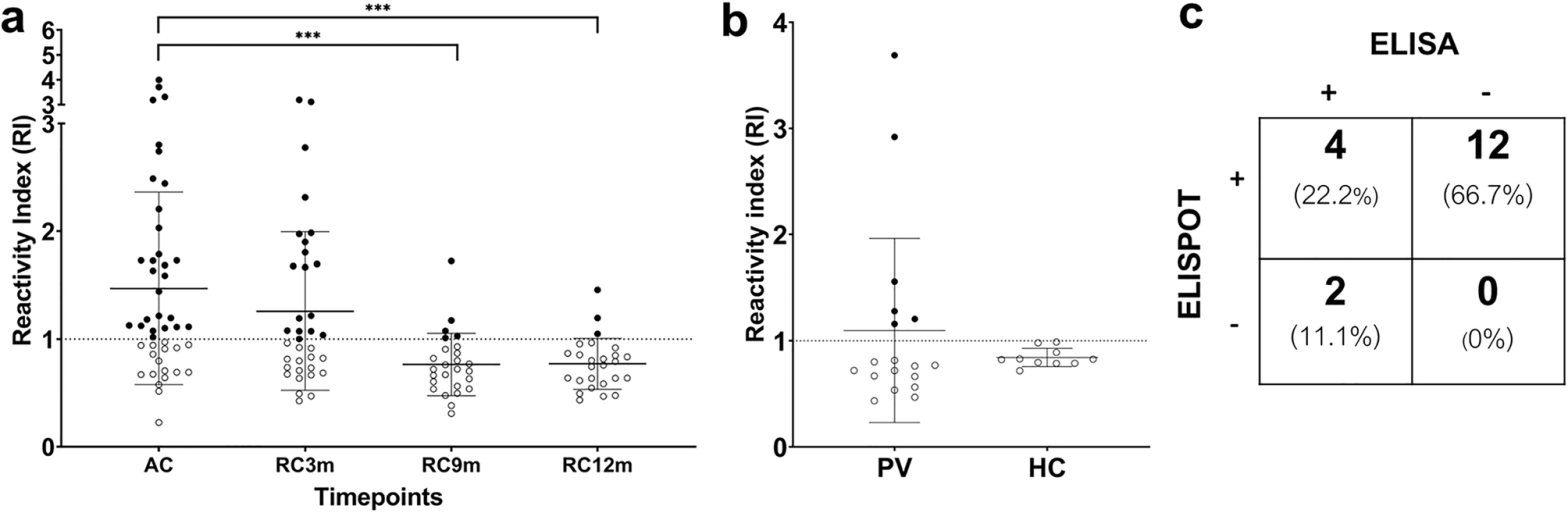
Antibody responses against recombinant PvDBPII protein in cross-sectional study 3. (**a**) Reactivity index (RI) against rPvDBPII protein at acute phase (AC, n = 45), 3 months (RC 3 m; n = 35), 9 months (RC 9 m; n = 26) and 12 months (RC 12 m; n = 26) post-infection. (**b**) Reactivity index (RI) of *P. vivax* infected subjects (n = 18) whose IFN-γ responses were determined by ELISPOT assay in cross-sectional study 1, compared to healthy donors (HCs, n = 10). (**c**) The correlation analysis between antibody and IFN-γ response to rPvDBPII protein in 18 *P. vivax* infected subjects whose samples were used for screening of potential peptide pools. The responses against peptide pool D were used for comparison due to the highest median and seropositivity percentage. Dashed horizontal line represents cutoff value for positive reactivity (RI = 1). Horizontal line represents mean ± SD. Statistical differences were tested by one-way ANOVA. ***p < 0.0001. *AC* acute, RC3m, RC9m and RC12m were 3, 9, 12 months recovery phase, respectively.

Since cooperation between T and B cells is important for development of protective immunity ^12^. Here, we assessed the antibody response to rPvDBPII protein during acute illness and after infection in *P. vivax* subjects in cross-sectional study 3. The seropositivity (RI > 1) was found at time of acute infection in 62% (28/45), and at 3-, 9- and 12-months post-infection in 51% (18/35), 19% (5/26) and 11% (3/26), respectively (Fig. 8a). To assess the correlation between antibody and IFN-γ responses to PvDBPII protein, the serological responses against this antigen were analysed in *P. vivax* subjects (n = 18) whose CD4^+^T cell responses were determined by ELISPOT assay in cross-sectional study 1. The result showed that 33% (6/18) of these subjects were positive (Fig. 8b). Further correlation analysis of seropositivity in individual subjects found that 22.2% (4/18) showed positive antibody and IFN-γ responses, 66.7% (12/18) displayed only IFN-γ responses and 11.1% (2/18) had only antibody response (Fig. 8c). Altogether, these results suggest that anti-PvDBPII antibody responses were produced and persisted in natural *P. vivax* infection and some subjects produced both antibody and CD4^+^T cell responses.

## Discussion

Memory CD4^+^T cells closely coordinate with B cells following infection and/or vaccination, sustaining protective immunity ^13–15^. Here, we analyzed MHC class II epitopes derived from blood-stage PvDBPII protein of *P. vivax* using in silico prediction and ex vivo characterization of T cell responses. Six dominant T cell epitopes capable of inducing the Th1 response of IFN-γ secretion were identified. These IFN-γ secreting cells showed effector memory T cell subset phenotype and a proportion of them were double cytokine positive (IFN-γ^+^ and TNF-α^+^) CD4^+^T cells. Of six T cell epitopes, three peptides containing polymorphic residues brought about altered levels of response in these IFN-γ producing cells. Analysis of antibody responses to PvDBPII peptides during acute malaria and after recovery revealed that some individuals had seropositive responses to the same peptides that drive memory T cell responses. Collectively, our data demonstrated that stimulation by PvDBPII epitopes could trigger responses by effector memory CD4^+^T cells following natural *P. vivax* infection.

Elucidation of the potential role of PvDBPII-specific cellular immunity will help in development of a protective *P. vivax* vaccine^16,17^. A previous study conducted in PNG identified potential epitopes located between cysteine regions (C4–C7) of PvDBPII capable of stimulating IFN-γ producing cell responses, supporting the promise of PvDBPII as a blood-stage antigen that can elicit T cell-mediated immunity ^6^. In the present study, we screened PvDBPII epitopes that bind to MHC class II molecules found frequently among Thais using in silico prediction ^8^. Peptides derived from the predicted T cell epitopes were examined for ability to stimulate responses in IFN-γ-producing T cells from subjects with *P. vivax* infection. Indeed, six potential PvDBPII T cell epitopes showed immunogenicity by triggering recall responses of memory CD4^+^T cells that developed during acute malaria and persisted after parasite clearance. These T cell epitopes were located in three clusters: cluster 1 (at residues 246–260), cluster 2 (residues 314–342) and cluster 3 (residues 435–449). Three of our potential epitopes were located in cluster 2. Peptide 5 (RDITFRKLYLKRKLI) overlapped by six amino acids with a previously identified epitope ^6^ and was located close to the DARC-PvDBPII binding site for reticulocyte invasion ^18^.Peptide 13 (GDLLLKLNNYRYNKD), located at residues 329–343, has exactly the same sequence as a previously characterized T cell epitope found in a PNG population ^6^. Of note, peptide 20 (SAIINHAFLQNTVMK), located in cluster 1, is first identified in this study. Together, our findings indicated hot spot regions of PvDBPII-derived T cell epitopes. Further experiments are needed to assess binding strength of T cell epitopes with MHC II complexes by in vitro functional assays and to evaluate immunogenicity of T cell epitopes, as well as to identify promiscuous T cell epitopes binding to multiple HLA alleles, to advance PvDBPII epitope-based vaccine development.

Long-term antigen-specific immunity in vivax malaria convalescents is crucial for protection upon re-exposure to *P. vivax* ^19^. Here, we first report the presence of PvDBPII-specific IFN-γ memory CD4^+^T cells along with their corresponding subsets following natural infection. The memory T cells were triggered by PvDBPII epitopes and these cells were maintained for at least two years after parasite clearance. The phenotypic analysis of these peptide-induced memory T cells revealed an effector memory T cell subset (CCR7^-^ CD45RA) that had IFN-γ/TNF-α co-producing properties. Our data was in line with previous studies showing the predominant responses of effector memory CD4^+^T cells during the acute or recovery phases of helminth or viral infections^20–24^. The protective function presence of these memory T cells in circulating blood was demonstrated by their migration to sites of infection and differentiation into effector Th1 and Th2 cells upon restimulation ^24^. To date, little is known about how effector memory CD4^+^T cells develop, are maintained and mediate protection against malaria infection. One possible mechanism explaining the development of memory CD4^+^T cells after PvDBPII stimulation may be that antigen is presented by antigen presenting cells (APCs) in lymphoid organs^25^. During cognate APC-CD4^+^T cell interaction, signals from APCs induce proliferation and activation of naïve CD4^+^T cells ^25^. These activated CD4^+^T cells differentiate into the effector Th1 cells, which produced IFN-γ and TNF-α cytokines, and drive development of memory cells ^26–28^. The polyfunctional of these effector PvDBPII-specific CD4^+^ T cells could help in malaria protection since it was reported that they increased durability of protection in malaria vaccine clinical studies ^29,30^. Our findings support the concept that effector memory CD4^+^T cells could persist in circulating blood following infection, poised to perform functional roles upon re-stimulation, including Th1 cytokine responses and provision of B cell help for antibody production.

Since the polymorphisms of PvDBPII arise from immune selection by altering the host immune responses to the variant residues ^22,23^, we examined the effect of mutations in T cell epitopes on cellular immunity to PvDBPII. Of six dominant T cell epitopes, three peptides contain polymorphic residues (R308S, L333F and W437R) which were identified in *P. vivax* endemic areas of Thailand and elsewhere ^24–26^. In this study, the effect of peptide polymorphism was tested in *P. vivax* subjects at early recovery phase (3–4 months). Residue 308S in the T cell epitope elicited lower responses in all studied *P. vivax* subjects, as compared to 308R. This might be attributable to the low prevalence of this *dbpII* polymorphism among Thai *P. vivax* isolates ^24^. For a single amino acid substitution at peptide 13, a leucine (L) to phenylalanine (F), only subjects RC02 and RC04 significantly responded to variant 333F. Similarly, subjects RC01 and RC04 exhibited significant responses to peptide 18 with 437R variant. The markedly increased responses to mutant T cell epitopes might be explained by those *P. vivax* subjects having been exposed to Thai PvDBPII variant strains (333F and 437R alleles), therefore generating naturally acquired cellular immunity to these variants. In contrast, subject RC03 had no response to *dbpII* variant alleles, indicating the mutation in PvDBPII generated variant-specific T cell responses. Moreover, there were differential responses to the same PvDBPII epitopes in each of the studied subjects who harbor a variety of MHC class II allotypes, suggesting there is genetic MHC restriction consistent with other disease models ^31–33^. Thus, the incorporation of these six T cell epitopes in a PvDBPII vaccine should be studied more in populations with different distributions of HLA II alleles.

It should be noted that our study has some limitations. First, this study lacked analysis of PvDBPII-specific T cell responses in acutely infected subjects, which precluded assessment of T cell responses to PvDBPII during infection. Second limitation was lack of kinetic analysis of IFN-γ CD4^+^T cell responses in individuals with full follow-up for 1–2 years after infection, which potentially could have explained the development and persistence of cellular immunity against DBPII. Third, HLA typing in a larger sample size of Thai *P. vivax* patients is required to represent PvDBPII-specific CD4^+^T cell responses in population and to demonstrate the effect of PvDBPII polymorphism on T cell responses. Since T cell responses are dependent on MHC interactions, further in vitro immunological investigation is needed to gain better insight into the interactions between PvDBPII peptides-MHC complexes, as well as the phenotypic and functional characteristics of PvDBPII-specific T cells.

In summary, we demonstrated the responses of PvDBPII-specific CD4^+^T cell following *P. vivax* infection. These specific CD4^+^T cells were present in the blood circulation with an effector memory cell phenotype and differentiated into a Th1 phenotype after restimulation. Knowledge regarding PvDBP epitope-induced T cell responses will help guide the development of improved peptide-based vaccines against vivax malaria.

## Supplementary Information

Supplementary Information.

Supplementary Table S6.

## Data Availability

All relevant data are within the manuscript and its supplementary information files.
