# Supplementary material for: Characterization of Duffy Binding Protein II-specific CD4+T cell responses in Plasmodium vivax patients
Source: Sci Rep. 2023 May 12;13:7741. doi: 10.1038/s41598-023-34903-4 (PMC10177721; doi:10.1038/s41598-023-34903-4)
Supplement: Supplementary file 1 — Supplementary Information. [file 41598_2023_34903_MOESM1_ESM.docx]

**Characterization of Duffy Binding Protein II-specific CD4^+^T cell responses in *Plasmodium vivax* patients**

Pongsakorn Thawornpan^1^, Chayapat Malee^1^, Piyawan Kochayoo^1^, Kittikorn Wangriatisak^1^, Chaniya Leepiyasakulchai, Francis B Ntumngia^2^, Sai Lata De^2^, John H. Adams^2^ and Patchanee Chootong^1*^

^1^Department of Clinical Microbiology and Applied Technology, Faculty of Medical Technology, Mahidol University, Bangkok, Thailand.
^2^Center for Global Health and Infectious Diseases Research and USF Genomics Program College of Public Health, University of South Florida, Tampa, Florida, USA.

* Corresponding author:
Patchanee Chootong, Ph.D.
Email: pchooton@gmail.com

**Supplementary Table S1. Demographic characteristics of *P. vivax* subjects and healthy subjects in cross-sectional study 1-2.**

| **Characteristics** | **Cross-sectional study 1** | | **Cross-sectional study 2** | |
| --- | --- | --- | --- | --- |
|  | **RC 3-24 months** | **Healthy controls** | **RC 1-2 months** | **RC 3-4 months** |
| Total Number | 18 | 10 | 7 | 4 |
| **Age (years)** | | | | |
| Median | 37 | 27 | 35 | 49 |
| (Q1, Q3) | (24, 46) | (26.0, 28.0) | (26.0, 49.0) | (34.8, 62.3) |
| **Gender** | | | | |
| Male | 72% (13/18) | 50% (5/10) | 71% (5/7) | 75% (3/4) |
| Female | 28% (5/18) | 50% (5/10) | 29% (2/7) | 25% (1/4) |

**Supplementary Table S2. Demographic characteristics of *P. vivax* subjects in cross-sectional study 3.**

| **Characteristics** | ***P. vivax* subjects** | | | | **Healthy controls** |
| --- | --- | --- | --- | --- | --- |
|  | **Acute** | **RC 3 months** | **RC 9 months** | **RC 12 months** |  |
| **Total Number** | 45 | 35 | 26 | 26 | 23 |
| **Age (years)** | | | | | |
| Median (Q1, Q3) | 32.0  (23.0, 41.0) | 28.0  (19.5, 37.0) | 28.0  (23, 42.5) | 27.5  (23.0, 37.8) | 24.0 (21.0, 27.0) |
| **Gender** | | | | | |
| Male | 67% (30/45) | 63% (22/35) | 69% (18/26) | 54% (14/26) | 57% (13/23) |
| Female | 33% (15/45) | 37% (13/35) | 31% (8/26) | 46% (12/26) | 43% (10/23) |
| **Nationality** | | | | | |
| Thai | 91% (41/45) | 74% (26/35) | 96% (25/26) | 96% (25/26) | 100% (23/23) |
| Myanmar | 9% (4/45) | 26% (9/35) | 4% (1/26) | 4% (1/26) | 0% (0/23) |
| **Parasitemia (parasite/µL)** | | | | | |
| Mean ± SD (Range) | 5150.03 ± 2522.20 (528.50 - 11608.19) | 0 | 0 | 0 | 0 |

**Supplementary Table S3. Amino acid sequence of PvDBPII used as input sequence for T cell epitope prediction by IEDB database.**

| **330 amino acid sequence of PvDBPII** |
| --- |
| TISSAIINHAFLQNTVMKNCNYKRKRRERDWDCNTKKDVCIPDRRYQLCMKELTNLVNNTDTNFHRDITFRKLYLKRKLIYDAAVEGDLLLKLNNYRYNKDFCKDIRWSLGDFGDIIMGTDMEGIGYSKVVENNLRSIFGTDEKAQQRRKQWWNESKAQIWTAMMYSVKKRLKGNFIWICKLNVAVNIEPQIYRWIREWGRDYVSELPTEVQKLKEKCDGKINYTDKKVCKVPPCQNACKSYDQWITRKKNQWDVLSNKFISVKNAEKVQTAGIVTPYDILKQELDEFNEVAFENEINKRDGAYIELCVCSVEEAKKNTQEVVTNVDNAA |

**Supplementary Table S4. List of 29 predicted PvDBPII-derived T cell epitope peptides in corresponding pools.**

| **Pool** | **Sequence** | **Peptide No.** |
| --- | --- | --- |
| **A** | DITFRKLYLKRKLIY | Peptide1 |
|  | ITFRKLYLKRKLIYD | Peptide2 |
|  | TFRKLYLKRKLIYDA | Peptide3 |
|  | FRKLYLKRKLIYDAA | Peptide4 |
|  | RDITFRKLYLKRKLI | Peptide5 |
|  | KLYLKRKLIYDAAVE | Peptide10 |
| **B** | MMYSVKKRLKGNFIW | Peptide8 |
|  | MYSVKKRLKGNFIWI | Peptide12 |
|  | YRWIREWGRDYVSEL | Peptide 18 |
|  | SAIINHAFLQNTVMK | Peptide 20 |
|  | RWIREWGRDYVSELP | Peptide 21 |
|  | SSAIINHAFLQNTVM | Peptide 22 |
| **C** | HRDITFRKLYLKRKL | Peptide7 |
|  | FHRDITFRKLYLKRK | Peptide9 |
|  | NFHRDITFRKLYLKR | Peptide 14 |
|  | KDIRWSLGDFGDIIM | Peptide 16 |
|  | DIRWSLGDFGDIIMG | Peptide 17 |
|  | IRWSLGDFGDIIMGT | Peptide 19 |
| **D** | RKLYLKRKLIYDAAV | Peptide6 |
|  | EGDLLLKLNNYRYNK | Peptide11 |
|  | GDLLLKLNNYRYNKD | Peptide 13 |
|  | VEGDLLLKLNNYRYN | Peptide 15 |
|  | LKRKLIYDAAVEGDL | Peptide 28 |
|  | LYLKRKLIYDAAVEG | Peptide 29 |
| **E** | IYRWIREWGRDYVSE | Peptide 23 |
|  | RWSLGDFGDIIMGTD | Peptide 24 |
|  | WIREWGRDYVSELPT | Peptide 25 |
|  | QIYRWIREWGRDYVS | Peptide 26 |
|  | KRKLIYDAAVEGDLL | Peptide 27 |

**Supplementary Table S5. Antibody fluorochromes for flow cytometry.**

| **Antibody** | **Manufacturer** | **Clone** | **Working dilution** |
| --- | --- | --- | --- |
| Zombie Green Fixable Viability Kit | Biolegend | - | 1/200 |
| Alexa Fluor 700 anti-human CD3 | Biolegend | OKT3 | 1/100 |
| PerCP/Cyaninne5.5 anti-human CD4 | Biolegend | SK3 | 1/200 |
| PE/Dazzle 594 anti-human CD45RA | Biolegend | HI100 | 1/100 |
| PE anti-human CD197 (CCR7) | Biolegend | G043H7 | 1/20 |
| PE/Cyanine7 anti-human IFN-γ | Biolegend | B27 | 1/50 |
| APC anti-human TNF-α | Biolegend | MAb11 | 1/100 |

**Supplementary Table S7.** **HLA genotyping results of 5 *P. vivax*-infected subjects recruited for cross-sectional study 1.**

| **Patient ID** | **HLA-DQA** | **HLA-DQB** | **HLA-DRB** | **IFN-γ SFUs/million PBMCs** | | | | | |
| --- | --- | --- | --- | --- | --- | --- | --- | --- | --- |
|  |  |  |  | **Peptide 6** | **Peptide 11** | **Peptide 13** | **Peptide 15** | **Peptide 28** | **Peptide 29** |
| RC01 | DQA1*01:01:01/DQA1*03:03:01 | DQB1*04:01:01/  DQB1*05:01:24 | DRB1*04:05:01**/** DRB1:15:02:01 | 249  (positive) | 145 | 297 (positive) | **2**24 (positive) | **2**35 (positive) | **3**30 (positive) |
| RC02 | DQA1*01:01:01/DQA1*01:05:01 | DQB1*05:01:01/  DQB1*05:01:01 | DRB1*01:01:01/  DRB1*10:01:01 | 199 (positive) | 140 | 197 (positive) | 150 | 192 (positive) | 240 (positive) |
| RC03 | DQA1*01:03:01/DQA1*03:02:01 | DQB1*03:03:02/  DQB1*05:03:01 | DRB1*04:05:01/  DRB1*09:01:02 | 111 | 322 (positive) | 182 (positive) | **2**15 (positive) | **2**77 (positive) | 366 (positive) |
| RC04 | DQA1*01:03:01/DQA1*06:01:01 | DQB1*03:01:01/  DQB1*06:01:01 | DRB1*08:03:02/  DRB1*12:02:01 | 242 (positive) | **261** (positive) | 331 (positive) | **170*** (positive) | **17**7* (positive) | 255 (positive) |
| RC05 | DQA1*01:04:01/DQA1*02:01:01 | DQB1*02:02:01/  DQB1*05:03:01 | DRB1*07:01:01/  DRB1*14:04:01 | 104 | **10**1 | 285 (positive) | **1**39 | **12**1 | 306 (positive) |

Positive response is defined as the number of spots in stimulated well are higher than cut off value calculated from doubling of mean plus 2SD of spots in non-stimulated wells of *P. vivax* infected subjects

*Two *P. vivax* samples show number of spots less than cut-off +1SD of mean non-stimulation (ns) were identified weakly positive and was not taken for HLA genotyping.

**Supplementary Table S8. HLA genotyping results of 3 *P. vivax*-infected subjects recruited for determining the effect of**

**PvDBPII peptide polymorphism in cross-sectional study 2.**

| **Patient ID** | **DQA** | **DQB** | **DRB** |
| --- | --- | --- | --- |
| RC01 | DQA1*01:01:01/DQA1*02:01:01 | DQB1*02:02:01/DQB1*05:01:24 | DRB1*07:01:01/DRB1*15:02:01 |
| RC02 | DQA1*01:01:01/DQA1*01:05:01 | DQB1*05:01:01/DQB1*05:01:01 | DRB1*01:01:01/DRB1*10:01:01 |
| RC04 | DQA1*01:01:01/DQA1*03:03:01 | DQB1*04:01:01/DQB1*05:01:24 | DRB1*04:05:01/DRB1:15:02:01 |

**Supplementary Figure S1. Representative INF-γ ELISPOT assay results.** Number of IFN-γ spot forming units (SFUs) per million PBMCs of *P. vivax* subjects after stimulation with 5 distinct peptide pools versus non-stimulation condition (NS). Positive IFN-γ responses to peptide pools were defined as responses in stimulated well that were higher than cut-off value calculated from doubling of mean plus 2SD of spots in non-stimulated wells of *P. vivax* infected subjects. Positive controls were prepared by stimulation of PBMCs with phytohaemagglutinin (PHA, 2% v/v), while negative controls were prepared by PBMCs without stimulation.

**
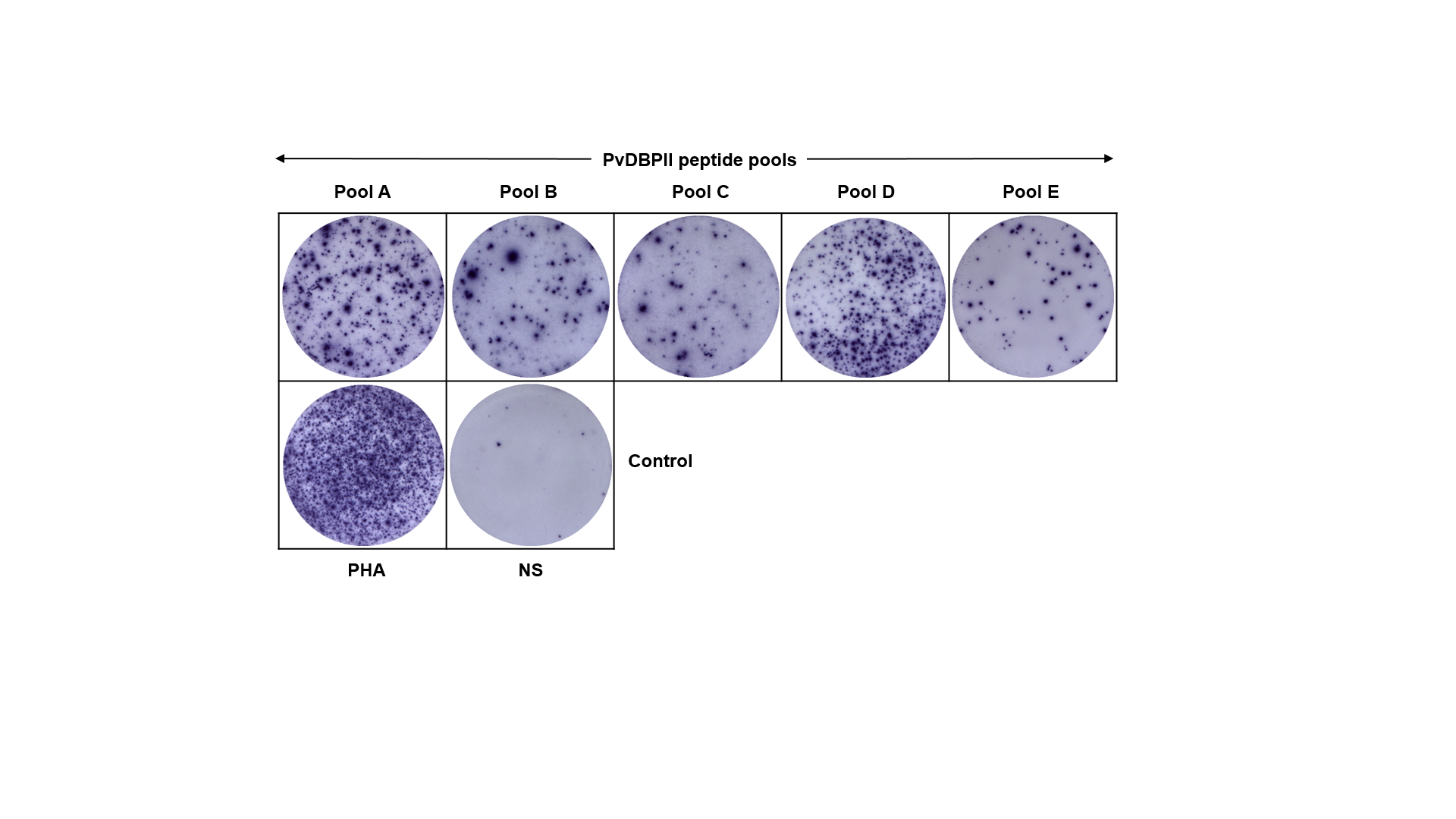
**
